# Rotational thromboelastometry-guided perioperative management of coagulation in a patient with Heyde’s syndrome undergoing transcatheter aortic valve implantation

**DOI:** 10.1186/s40981-019-0224-3

**Published:** 2019-01-11

**Authors:** Kumi Fukuhara, Takashi Kondo, Hirotsugu Miyoshi, Hiroshi Hamada, Masashi Kawamoto

**Affiliations:** Department of Anesthesiology and Critical Care, Hiroshima University Hospital, 1-2-3 Kasumi, Minami-ku, Hiroshima 734-8551 Japan

**Keywords:** Heyde’s syndrome, Rotational thromboelastometry, Transcatheter aortic valve implantation

## Abstract

**Background:**

Changes in coagulability during the hyperacute phase within 24 h after transcatheter aortic valve implantation (TAVI) for Heyde’s syndrome, or aortic stenosis complicated by gastrointestinal angiodysplasia and acquired coagulation dysfunction, have not been clarified. We evaluated perioperative changes in coagulability using rotational thromboelastometry (ROTEM).

**Case presentation:**

A female patient with Heyde’s syndrome in her 80s underwent TAVI. ROTEM showed coagulation dysfunction before and at 6 h after surgery. Improvements in coagulation function started at 12 h after surgery. Based on ROTEM findings, oral administration of antiplatelet agents was started on the day after surgery. No hemorrhagic complications were observed in the postoperative phase.

**Conclusions:**

Evaluation of coagulation function using ROTEM was useful for monitoring perioperative hemostasis and coagulation in this patient.

## Background

Heyde’s syndrome, or aortic stenosis (AS) complicated by gastrointestinal hemorrhage attributed to gastrointestinal angiodysplasia and acquired coagulation dysfunction, is reported to affect approximately 20% of patients with severe AS [1–3]. In Heyde’s syndrome, blood flow is accelerated and shear stress is generated when passing through the stenotic aortic valve, which leads to destruction and deficiency of high-molecular-weight vWF multimers. This results in a primary hemostatic disorder classified as acquired von Willebrand syndrome type 2A (AVWS-2A) [3]. Normally, vWF binds to coagulation factor VIII and forms a stable inactive complex. In AVWS-2A, clot formation is impaired by dysfunctional interaction between platelets and the blood coagulation system even with normal factor VIII activity and the absence of abnormalities in PT or APTT [4, 5].

Rotational thromboelastometry (ROTEM; TEM international GmbH, Munich, Germany), a coagulation monitoring method using whole blood at the bedside, can easily evaluate blood coagulation abnormalities that cannot be detected by tests of plasma [6, 7]. In the EXTEM assay, activation of the extrinsic blood coagulation pathway by tissue factor is evaluated. In the INTEM assay, activation of the intrinsic blood coagulation pathway by contact coagulation activators is evaluated. Moreover, in the FIBTEM assay, abnormalities in platelet function and decreased capability for fibrin polymerization caused by impairment of coagulation factors can be differentiated by evaluating the capability for fibrin polymerization when platelet aggregation is inhibited by a platelet aggregation inhibitor and the extrinsic coagulation pathway is stimulated by tissue factor [6–8].

Although treatment for AS has been shown to ameliorate Heyde’s syndrome [2], no study has evaluated changes in coagulability over time during the hyperacute phase within 24 h after surgery. We describe the perioperative management of a patient with severe AS diagnosed with Heyde’s syndrome undergoing transcatheter aortic valve implantation (TAVI). We used ROTEM to monitor hemostasis and coagulation in order to assess changes in coagulability over time. The timing of postoperative treatment with antiplatelet agents was determined based on ROTEM results.

## Case description

**Fig. 1:**
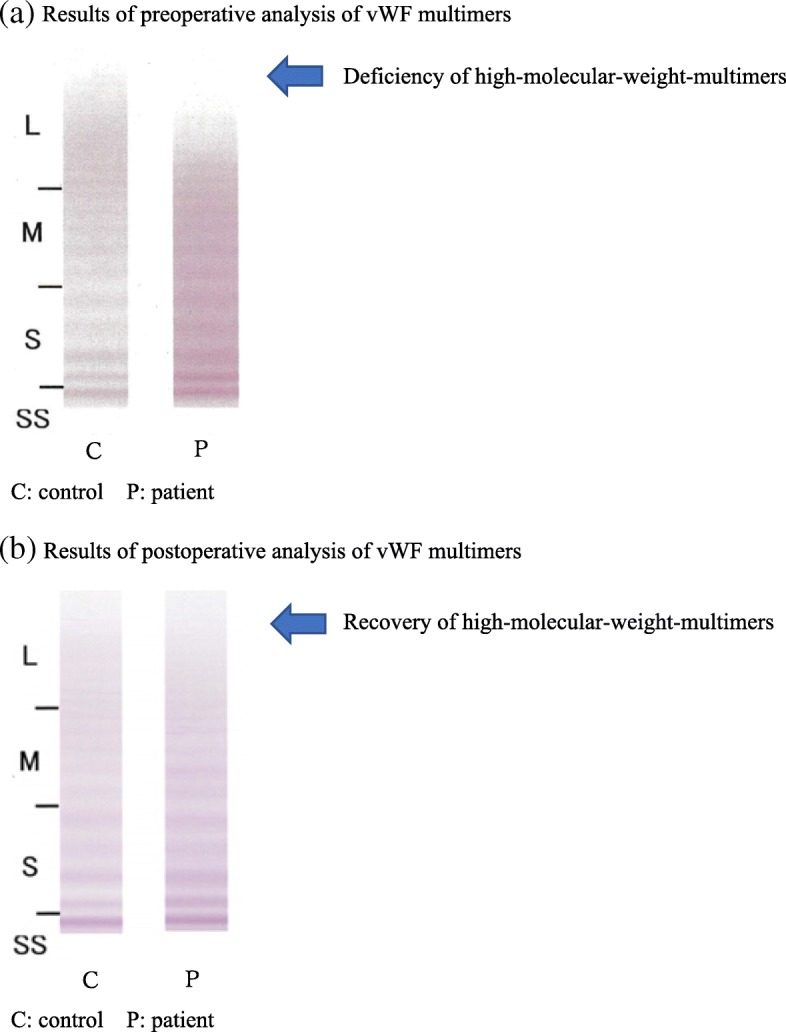
**a** Results of preoperative analysis of vWF multimers. **b** Results of postoperative analysis of vWF multimers

A female patient with AS in her 80s had recurrent gastrointestinal hemorrhage, epistaxis, and submucosal hemorrhage during the course of AS. She was 140 cm tall and weighed 45 kg. Transthoracic echocardiography indicated severe AS with an aortic valve area of 0.55 cm^2^ (trace) and maximum blood flow rate of 6.8 m/s, and mild aortic valve regurgitation. Left ventricular ejection fraction was 65%. No left ventricular wall motion abnormalities were observed. Coronary angiography did not show any significant stenosis. Blood tests indicated anemia (hemoglobin 11.8 g/dL) and thrombocytopenia (platelet count, 8.0 × 10^4^/μL). Prothrombin time (PT) and activated partial thromboplastin time (APTT) were within the normal range, while vWF analysis indicated deficiency of high-molecular-weight multimers (Fig. 1a). Based on these findings, TAVI was scheduled given the patient’s condition. Oral administration of carbazochrome sulfonic acid and tranexamic acid were added prior to surgery, then the bleeding tendency was improved.

General anesthesia was induced with intravenous midazolam (3 mg), fentanyl (100 μg), and rocuronium (50 mg). After tracheal intubation, anesthesia was maintained with propofol (0.5 μg/ml of target control infusion [TCI]) and remifentanil (0.2 μg/kg/min). A prosthetic valve was placed using the transfemoral approach after 4500 units of heparin were administered. Intraoperative respiration and hemodynamics were stable. After placing the prosthetic valve, 45 mg of protamine were administered and the operation was completed. Intraoperative blood loss was 214 ml without apparent bleeding symptoms, then 900 ml of crystalloid and 500 ml of colloid were infused. After the patient recovered from anesthesia, she was transferred to the intensive care unit.

Tables 1 and 2 shows the results of perioperative laboratory and ROTEM findings. ROTEM clearly indicated coagulation dysfunction preoperatively and at 6 h after surgery, which improved starting at 12 h after surgery. Coagulation testing showed that APTT was slightly increased from immediately to 24 h after surgery and normalized on the third postoperative day. Based on ROTEM findings, oral administration of antiplatelet agents was started on the day after surgery. Subsequently, no bleeding tendency was observed clinically. On the day after surgery, vWF analysis showed recovery of high-molecular-weight multimers (Fig. 1b). Transthoracic echocardiography indicated decreased AS (mean aortic valve systolic pressure gradient, 14 mmHg). The patient was discharged in good condition.

**Table 1:** Blood testing findings

	Before surgery	6 h after surgery	24 h after surgery	3 days after surgery
Plt (× 10^3^/μL) [150–360]	80	63	61	69
PT-INR [0.85–1.15]	1.02	1.1	1.07	1.05
APTT (s) [26.9–38.1]	32.9	38.8	39.7	35
Fib (mg/dL) [200–400]	225.6	189.5	239.9	319.2

*Plt* platelet count, *PT-INR* prothrombin time-international normalized ratio, *APTT* activated partial thromboplastin time, *Fib* fibrinogen

**Table 2:** ROTEM findings

	Before surgery	6 h after surgery	12 h after surgery	24 h after surgery	3 days after surgery
EXTEM					
CT (s) [38–79]	59	50	54	57	51
CFT (s) [34–159]	200	130	123	131	103
A10 (mm) [43–65]	35	44	44	44	49
MCF (mm) [50–72]	44	52	52	53	56
INTEM					
CT (s) [100–240]	308	403	191	240	157
CFT (s) [30–110]	190	182	123	127	102
A10 (mm) [44–66]	38	37	43	44	47
MCF (mm) [50–72]	47	46	51	51	52
FIBTEM					
A10 (mm) [7–23]	13	14	–	13	16
MCF (mm) [9–25]	14	15	–	14	19

Units are given in parentheses and reference ranges are given in square brackets

*ROTEM* rotational thromboelastometry, *EXTEM* tissue factor reagent, *INTEM* contact coagulation activator reagent, *FIBTEM* modified EXTEM test with a platelet aggregation inhibitor, *CT* clotting time, *CFT* clot formation time, *A10* amplitude at 10 min, *MCF* maximum clot firmness

## Discussion

Coagulopathy in Heyde’s syndrome is expected to abate when AS is treated and destruction of high-molecular-weight vWF multimers stops. With both surgical aortic valve replacement and TAVI, recovery of high-molecular-weight vWF multimers has been reported on the day after surgery [2, 9, 10]. Although vWF multimer analysis is used as a standard diagnostic method for AVWS-2A associated with Heyde’s syndrome [11], it is time-consuming and therefore not suitable for chronological evaluation of changes in coagulability during the acute postoperative phase. By measuring PT and APTT, abnormalities in extrinsic and intrinsic coagulation factors are assessed using plasma; neither platelet function, including its interaction with the blood coagulation system, nor the firmness of blood clots can be evaluated [8]. Compared with plasma-based tests, we believe that ROTEM, which monitors the coagulation system using whole blood, can comprehensively and promptly evaluate coagulation dysfunction not reflected by PT or APTT. Thus, we selected ROTEM for perioperative monitoring of hemostasis and coagulation in our patient.

ROTEM clearly indicated coagulation dysfunction preoperatively and at 6 h after surgery, which decreased over time starting at 12 h after surgery in this patient. Although oral antiplatelet agents commonly used for the prevention of embolism after TAVI would be difficult to administer when coagulation dysfunction remains or postoperative gastrointestinal hemorrhage occurs [12], we were able to start oral antiplatelet agents based on improvements in coagulation function on the day after surgery indicated by ROTEM. Consequently, the patient was managed without hemorrhagic complications.

During the preoperative ROTEM evaluation in this patient, decreases in EXTEM-maximum clot firmness (MCF) and INTEM-MCF were observed. FIBTEM-MCF was within the normal range. MCF reflects the maximum firmness of the clot ultimately formed by the interaction between platelets and polymerized fibrin. The ROTEM results for MCF indicated a normal capability for fibrin polymerization and decreased platelet function in our patient. Preoperative increases in EXTEM-clot formation time (CFT), INTEM-CFT, and INTEM-clotting time (CT) in this patient indicate prolongation of the time to clot formation [13], which suggests dysfunction of the coagulation system, specifically the part involving intrinsic and extrinsic coagulation factors. Because PT and APTT measured in blood tests were within the normal range, the above changes were considered to be either the result of abnormalities in blood coagulation pathways other than the coagulation process reflected by PT or APTT or the result of abnormalities in clot formation caused by impaired interaction between platelets and the blood coagulation system associated with AVWS-2A.

Because ROTEM can assess abnormalities in platelet function, its results can serve as a guide to whether platelet transfusion is necessary [13]. As is the case with abnormal platelet function, thrombocytopenia impairs clot formation and thus can affect ROTEM findings [14]. However, improvements in ROTEM findings despite a postoperative decrease in platelet count in our patient suggest that thrombocytopenia was not the main cause of preoperative coagulation dysfunction in this patient. In ROTEM, adhesion of platelets to damaged vascular endothelium mediated by vWF in the physiological environment cannot be reproduced; therefore, direct evaluation of abnormal vWF function is speculated to be difficult [13]. Nevertheless, in this patient, abnormal ROTEM findings were observed despite the absence of clear PT or APTT prolongation after surgery, and postoperative improvement in ROTEM findings over time and recovery of high-molecular-weight multimers of vWF were noted. These findings suggest that both coagulation disorders mediated by abnormal vWF function associated with AVWS-2A and impaired interaction between platelets and the blood coagulation system were occurring before surgery, and that ROTEM findings reflected the overall recovery of coagulability achieved by aortic valve replacement and resulting resolution of Heyde’s syndrome.

We used ROTEM to monitor hemostasis and coagulation in the perioperative management of a patient with Heyde’s syndrome undergoing TAVI and assessed changes in coagulability over time. Based on ROTEM findings indicating improvement, oral antiplatelet agents were started and the patient was managed without hemorrhagic complications. In the treatment of Heyde’s syndrome using TAVI, when coagulation dysfunction not detected by common testing methods continues during the acute postoperative phase, the risk of hemorrhagic complications is increased and the use of oral antiplatelet agents is difficult. For this reason, comprehensive evaluation of coagulability by ROTEM might be a useful method to monitor perioperative hemostasis and coagulation.

## Abbreviations

APTT: Activated partial thromboplastin time
AS: Aortic stenosis
AVWS-2A: von Willebrand syndrome type 2A
CFT: Clot formation time
CT: Clotting time
MCF: Maximum clot firmness
PT: Prothrombin time
ROTEM: Rotational thromboelastometry
TAVI: Transcatheter aortic valve implantation
